# Paleoenvironmental and paleobiogeographical significance of Paleocene – early Eocene ostracods in Wadi Tarfa, North Eastern Desert, Egypt

**DOI:** 10.1038/s41598-025-89560-6

**Published:** 2025-02-25

**Authors:** Ahmed Samir, El Sayed M. Moneer, Islam El-Sheikh, Youssef S. Bazeen

**Affiliations:** 1 Geology Department, Faculty of Science, Al-Azhar University, PO. Box: 11884, Cairo, Egypt; 2 Geology Department, Faculty of Science, Al-Azhar University, Assiut Branch, PO. Box: 71524, Assiut, Egypt

**Keywords:** Ostracoda, Paleocene-early Eocene, Eastern Desert, Biostratigraphy, Paleobathymetery, Tythian affinity, Stratigraphy, Palaeontology, Sedimentology

## Abstract

This study investigates ostracod faunas from the well-preserved Paleocene to lower Eocene sedimentary succession at Wadi Tarfa, North Eastern Desert, Egypt. A total of 22 species and subspecies across 16 genera and 8 families were identified across 57 samples. Three zones were identified: *Doricythereis jordanica jordanica* Zone, *Cytheropteron toshkaensis* Zone, and *Phalcocythere horraensis* Zone, based on the stratigraphic distribution of ostracod fauna. However, correlations with other sections revealed inconsistencies in the first and last occurrences of ostracod species, indicating complexities in regional biostratigraphic correlation by ostracod fauna and the influence of localized depositional factors. Both R-mode and Q-mode clustering analyses were applied to ostracod assemblages, identifying four distinct faunal clusters and five biofacies, reflecting depositional changes from outer neritic to upper bathyal environments. The late Paleocene revealed reduced ostracod diversity, correlating with a deepening marine environment, particularly in the Tarawan Formation. Non-Metric Multidimensional Scaling (NMDS) analyses indicated significant paleobiogeographic connections between North African and Levantine sites, while faunal differentiation was greater in West African and Middle Eastern regions due to marine barriers.

## Introduction

The Paleocene-lower Eocene succession in Egypt is characterized by variations in the vertical and horizontal stratigraphic distribution, environmental changes, and significant climatic changes, particularly during the Paleocene-Eocene Thermal Maximum (PETM). This period, occurring about 56 million years ago, exhibited a swift global warming by 5–8 °C, which had profound effects on the sedimentary records and the associated faunal assemblages in Egypt^1^.

The well-developed Paleocene–lower Eocene succession represents an important part of the stratigraphic succession and has garnered considerable interest from numerous researchers over the years. Numerous studies have been conducted on the Paleocene-Eocene succession in Egypt, focusing on their lithostratigraphy, biostratigraphy, paleoecology, chemostratigraphy, and paleobiogeography^2–6^. These studies helped in understanding the global changes that occurred during the Paleocene-Eocene transition.

Despite numerous studies concerned with the Paleocene-early Eocene ostracoda in and outside of Egypt have been published^7–26^, only one ostracod paper was published on the ostracods of the Wadi Tarfa section^27^, which focused on the taxonomy and paleobathymetric changes in the Southern Galala Plateau, Eastern Desert.

In this study, we seek to enhance the knowledge of the ostracod faunal composition during the Paleocene-early Eocene time in the Wadi Tarfa section, North Eastern Desert and inspect them for their biostratigraphic, paleoenvironmental, and paleobiogeographical implications. The Wadi Tarfa section contains a well-preserved succession of Paleocene-lower Eocene marine sediments, making it an ideal site for investigating ostracod community responses to environmental shifts during this period.

## Geologic setting

**Fig. 1:**
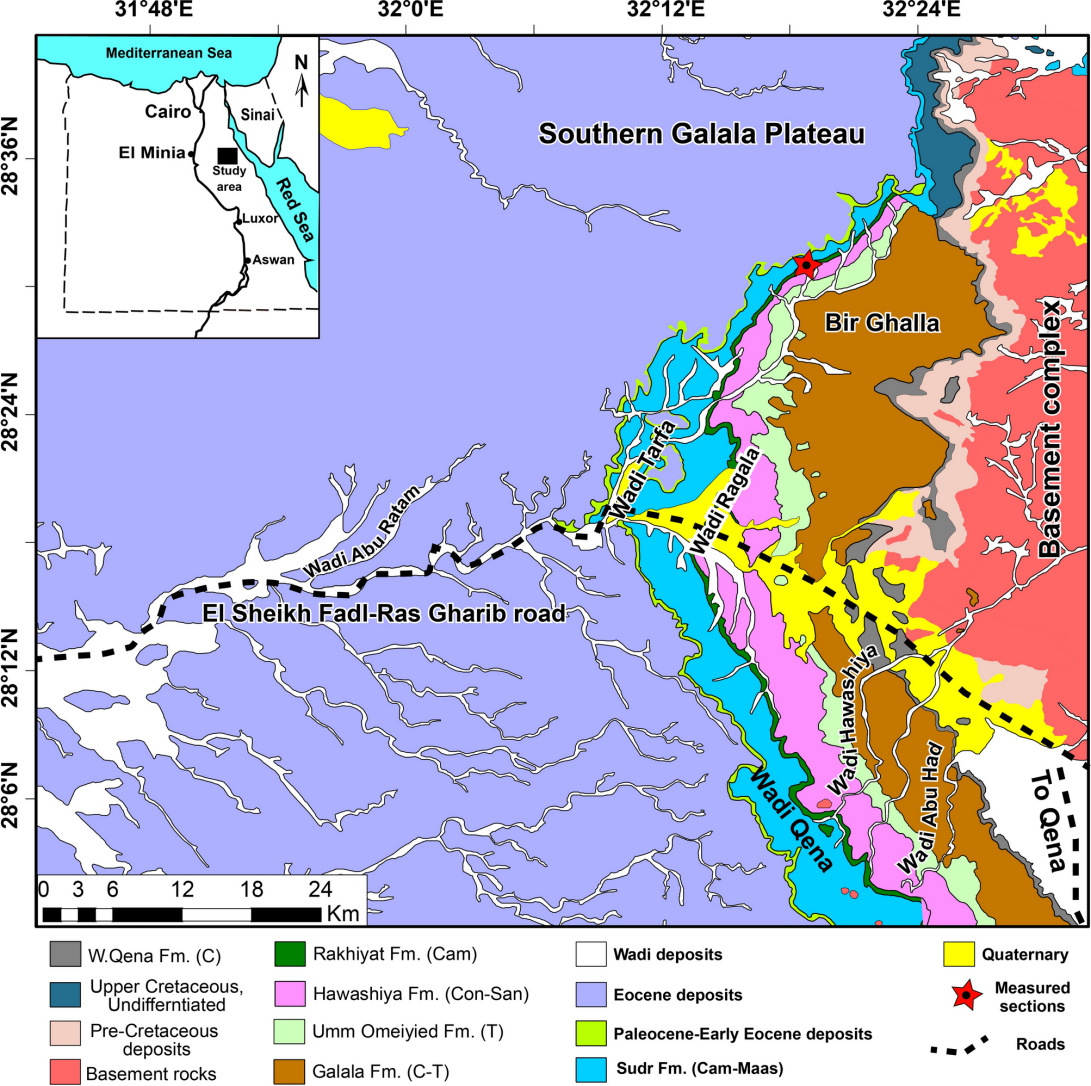
Geological map of the area under study. The map was derived from the geological map of Egypt, Beni Suef sheet (simplified after Klitszch et al.^31^). The abbreviations in parentheses indicate the age; Cenomanian (C), Turonian (T), Coniacian (Con), Santonian (San), Campanian (Cam), Maastrichtian (Maas).

The area under study is positioned in Wadi Tarfa, which is situated in the North Eastern Desert, Egypt (Fig. 1) within the southern passive continental margin of the vast Tethyan Ocean. During this time, the area underwent a major marine transgressive cycle as sea levels rose substantially worldwide^28^. Consequently, the shoreline of the Tethys Ocean expanded significantly southward, flooding the Northern African craton, which forms much of Egypt’s bedrock geology. This enormous marine inundation led to widespread deposition across the country, with the Tethys coastline extending over 1000 km south of its current position^29^. The regional paleogeography was transformed, as North Egypt became covered by extensive deeper-water paleoenvironments. As a result, thick sedimentary strata from the Upper Cretaceous to Lower Paleogene periods were deposited across the region, currently covering approximately 62% of the Egyptian surface area^30^.

The Syrian Arc System, a regional compressional event, also impacted the geography of North Egypt during the Late Cretaceous-Early Paleogene. Uplift along the Syrian Arc created differentiated paleotopographic features with localized basins and swells. For example, the Galala Mountains, north of the studied area, represent the southern branch of the Syrian Arc compressional uplift^29,32–34^. Together with sea level fluctuations, this orogenic activity controlled facies and paleontological patterns in the Upper Cretaceous-Lower Paleogene strata^29,35^.

The Upper Cretaceous-Lower Paleogene succession rests unconformably over the Naqus Formation (non-marine Paleozoic strata), which denotes the oldest sedimentary unit within the study area. The section comprises Upper Cretaceous units like the Galala, Umm Omeiyed, Hawashiya, Rakhiyat, and Sudr formations. The lithofacies gradient of these Upper Cretaceous units reflects deposition in progressively deeper neritic settings^36^. This grades upward into Paleocene to Eocene units, including the Dakhla, Tarawan, Esna, and Thebes formations (Fig. 1), recording maximum transgression and eventual carbonate platform development. These formations reach hundreds of meters in total thickness across North Eastern Desert of Egypt. They provide a detailed record of paleoceanographic changes along the ancient Tethyan margin, including relative sea level rises, shifting coastal geomorphology, and evolution of marine ecosystems from the late Mesozoic into the early Cenozoic^34,35,37^. Careful study of sections like that preserved in Wadi Tarfa yields critical insights into the geologic history of passive margin sedimentation, paleoenvironmental parameters, and biotic communities along the southern Tethyan shoreline, which once extended across the now barren deserts of the North Eastern Desert.

**Fig. 2:**
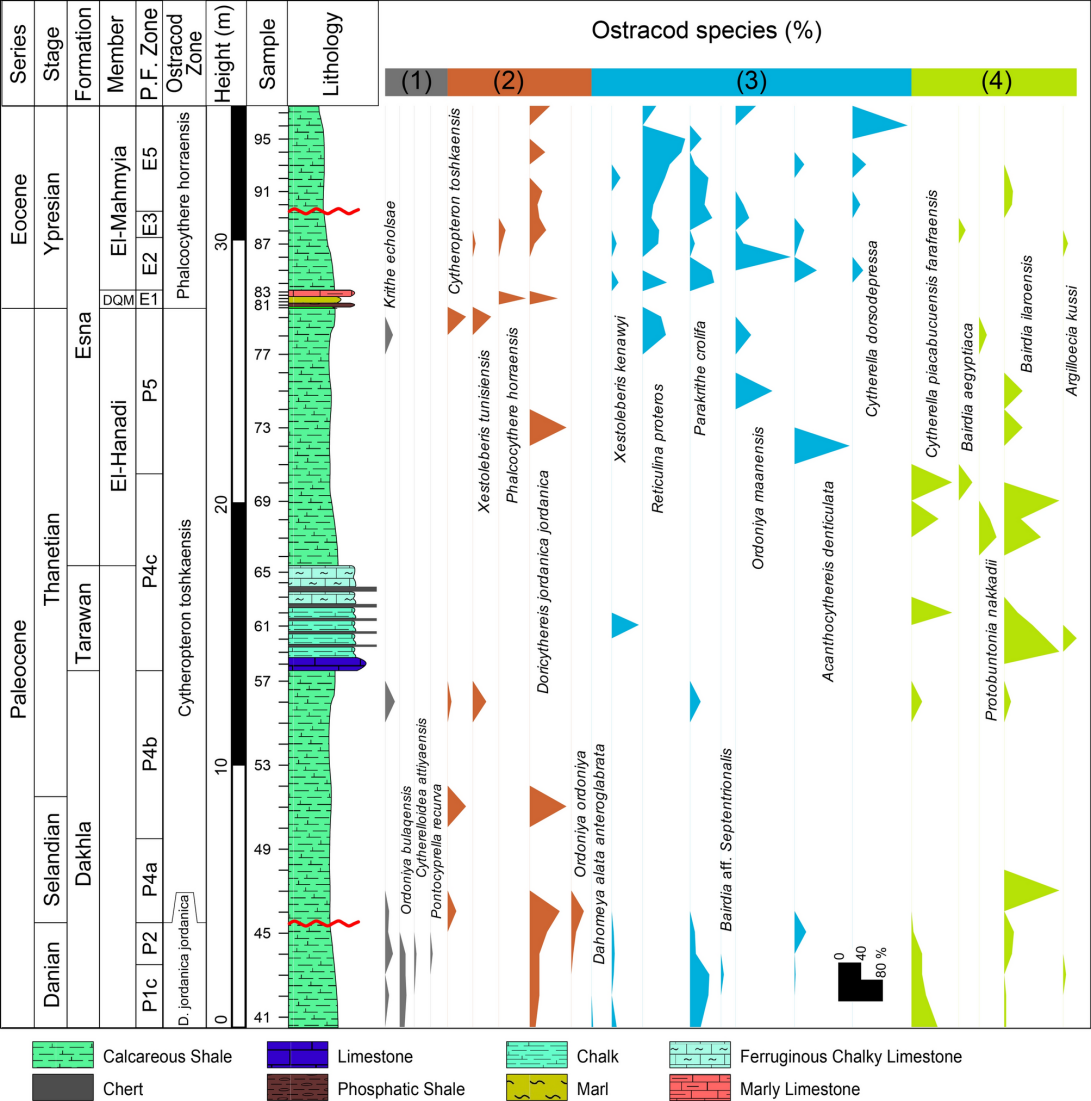
Stratigraphic succession of the studied Paleocene-early Eocene section, including the biostratigraphic zonation and the distribution of the ostracod species that arranged based on the R-mode clustering.

In this study, we are concerned with the Paleocene to Eocene units, including the Dakhla, Tarawan, and Esna formations. The Dakhla Formation consists of gray calcareous shale and marl with approximately 13.6 m total thickness. The Tarawan Formation follows up with an unconformable relationship and appears as a prominent 3 m thick ledge of chalk interbedded with repeated chert bands. The chalk is harder in the lower portion and eventually becomes more ferruginous and softer toward the upper portion. The Esna Formation in the study section comprises three distinct members. The El-Hanadi Member (9 m thick) is of Paleocene (Thanetian) age and composed of grey to green, slightly compact calcareous shale and marl. The Dababyia Quarry Member (DQM) follows up with a total thickness of about 70 cm and comprises three distinctive beds: dark brown phosphatic shale, pale yellow marl, and light grey argillaceous limestone. The DQM is known to designate the onset of the Eocene Epoch across different Egyptian provinces^38–40^. The El-Mahmiya Member comprises grey, fissile, calcareous shales measuring approximately 7 m thick. It represents the upper part of the Esna Formation and is capped by the limestones of the overlying Thebes Formation (Fig. 2).

## Material and methods

**Fig. 3:**
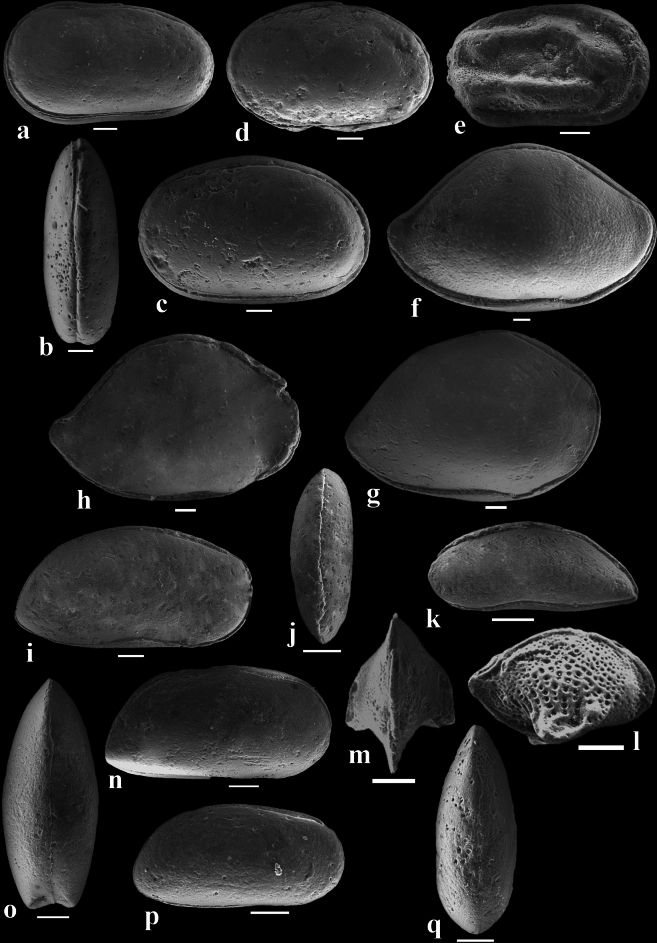
SEM micrographs of selected ostracod species. HRV: right view, LV: left view, DV: dorsal view, L: length, H; height, W: width. Scale bar = 100 µm. (**a**), (**b**) *Cytherella dorsodepressa* Morsi, Hewaidy and Samir, 2019. sample 93: a, WT-01, L. 0.88, H. 0.49, LV; b, WT-02, L. 0.81, W. 0.30, DV. (**c**), (**d**) *Cytherella piacabucuensis farafraensis* Bassiouni and Morsi, 2000. sample 41: 3, WT-03, L. 0.89, H. 0.58, LV; 4, WT-04, L. 0.78, H. 0.49, LV. (**e**) *Cytherelloidea attiyaensis* Morsi, 1999. sample 43, WT-05, L. 0.67, H. 0.40, LV. (**f**) *Bairdia aegyptiaca* Bassiouni and Morsi, 2000. sample 88, WT-06, L. 1.53, H. 0.97, RV. (**g**) *Bairdia ilaroensis* Reyment and Reyment, 1959. sample 44, WT-07, L. 1.15, H. 0.8, RV. (**h**) *Bairdia* aff. *septentrionalis* Bonnema, 1941. sample 43, WT-08, L. 1.21, H. 0.80, RV. (**i**) *Pontocyprella recurva* Esker, 1968. sample 44, WT-09, L. 0.89, H. 0.45, RV. (**j**), (**k**) *Argilloecia kussi* Bassiouni and Morsi, 2000. sample 43: (**j**), WT-10, L. 0.45, W. 0.18, DV; k, WT-11, L. 0.48, H. 0.19, LV. (**l**), (**m**) *Cytheropteron toshkaensis* Bassiouni and Luger, 1990. l, sample 46: WT-12, L. 0.40, H. 0.25, RV; m, sample 79: WT-13, L. 0.37, W. 0.29, DV. (**n**), (**o**) *Krithe echolsae* Esker, 1968. sample 56: n, WT-14, L. 0.73, H. 0.38, RV; o, WT-15, L. 0.71, W. 0.30, DV. (**p**), (**q**) *Parakrithe crolifa* Bassiouni and Luger, 1990. p, sample 85: WT-16, L. 0.56, H. 0.31, RV; q, sample 92: WT-17, L. 0.54, W. 0.22, DV.

**Fig. 4:**
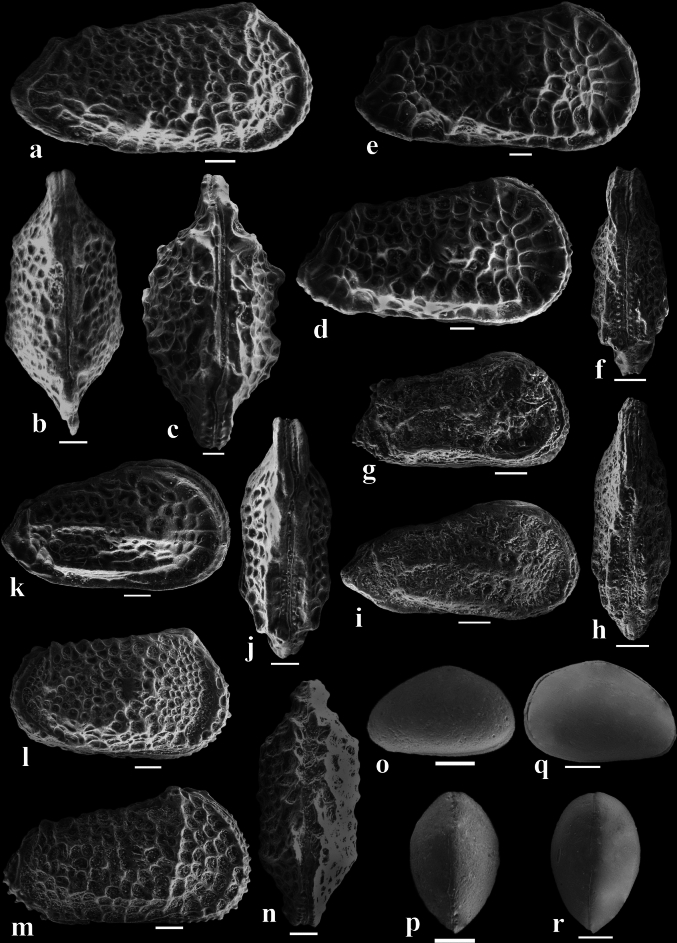
SEM micrographs of selected ostracod species. HRV: right view, LV: left view, DV: dorsal view, L: length, H; height, W: width. Scale bar = 100 µm. (**a**), (**b**) *Acanthocythereis denticulata* Esker, 1968. sample 85: a, WT-18, L. 0.99, H. 0.49, RV; b, WT-19, L. 0.94, W. 0.42, DV. (**c**), (**e**) *Doricythereis jordanica jordanica* (Bassiouni, 1970). sample 88: c, WT-20, L. 1.21, W. 0.64, DV; d, WT-21, L. 1.14, H. 0.61, RV; e, WT-22, L. 1.25, H. 0.65, RV. (**f**), (**g**): *Ordoniya ordoniya* (Bassiouni, 1970). sample 45: f, WT-23, L. 0.65, W. 0.25, DV; g, WT-24, L. 0.67, H. 0.36, RV. (**h**), (**i**) *Ordoniya bulaqensis* Bassiouni and Luger, 1990. sample 43: (**h**) WT-25, L. 0.74, W. 0.25, DV; (**i**) WT-26, L. 0.75, H. 0.38, RV. (**j**), (**k**) *Ordoniya maanensis* (Bassiouni, 1970). sample 89: (**j**) WT-27, L. 0.85, W. 0.35, DV; k, WT-28, L. 0.76, H. 0.35, RV. (**l**) *Phalcocythere horraensis* Bassiouni and Morsi, 2000*.* sample 88, WT-29, L. 0.83, H. 0.46, RV. (**m**), (**n**) *Reticulina proteros* Bassiouni, 1969. sample 88: m, WT-30, L. 0.90, H. 0.49, RV; n, WT-31, L. 0.88, W. 0.38, DV. (**o**), (**p**) *Xestoleberis kenawyi* Khalifa and Cronin, 1979. sample 41: o, WT-32, L. 0.38, H. 0.24, LV; p, WT-33, L. 0.36, W. 0.23, DV (**q**), (**r**) *Xestoleberis tunisiensis* Esker, 1968. q, sample 79: WT-34, L. 0.43, H. 0.29, RV; m, sample 56: WT-35, L. 0.41, W. 0.27, DV.

The investigated ostracod material is derived from 57 rock specimens obtained from the Paleocene-lower Eocene succession of the Wadi Tarfa section, North Eastern Desert of Egypt. For each sample, approximately 100 g of dried rock was crushed, treated with hydrogen peroxide (15%). A day after, the mixture was washed through a 0.063 mm sieve. The residue was dried, then sieved, allowing for the extraction, identification, and counting of all ostracods present in the residue. Most of the ostracod species identified were photographed by Scanning Electron Microscope (SEM) in the laboratories of the Egyptian Desert Research Center (DRC) for detailed morphological analysis (Figs. 3 and 4). The species described here are stored at the Geology Department, Faculty of Science, Al-Azhar University (Cairo, Egypt), with reference numbers WT01-WE35. To detect the paleoecological variances within the examined section, two-way hierarchical clustering analyses were performed on the ostracod species relative distribution data using the web-based ClustVis tool, applying correlation distance and Ward linkage^41^. These methods were chosen because the ostracod data exhibited substantial variability between samples in a quantitative context. The combination of Ward linkage and correlation distance was considered optimal for minimizing within-cluster variance and effectively accommodating high data variability, thereby highlighting meaningful ecological gradients and patterns. Both Q-mode (samples-based) and R-mode (species-based) clustering approaches were applied to identify patterns in species abundance and sample grouping. The results were visualized through dendrograms and a heatmap to highlight species groupings and ecological differences across the sampled section. The geographic distributions of the recognized ostracod species were compiled based on relevant literature and analysed through a non-metric multidimensional scaling (NMDS) with PAST package^42^ to assess their paleobiogeographic patterns. NMDS was applied to a species presence/absence matrix across different regions, with correlation used as the similarity measure. The resulting stress value attained 0.2256, indicating that the ordination does not fully capture the associations between ostracod faunas in each region. The coefficient of determination (R^2^) for the first and second axes were 0.3667 and 0.2784, respectively, suggesting that while the analysis provides some insight into the biogeographic relationships, the results should be interpreted with caution. The NMDS technique was applied to visualize similarities and dissimilarities in ostracod community composition across different geographical regions, thereby identifying potential biogeographical clustering and distribution patterns.

## Result

### Systematic paleontology

The ostracod fauna examined in this study has been taxonomically categorized into 22 species and subspecies belonged to 16 genera and 8 families. The classification framework employed follows the guidelines set by Horne^43^. Generic assignments primarily adhere to Moore^44^, with later-established genera considered based on the proposals of their respective authors. SEM images of the recorded ostracod taxa were demonstrated in Figs. 3 and 4. The taxonomic list can be found in Appendix 1.

### Biostratigraphy

The examined ostracod material was obtained from a stratigraphic succession spanning early Paleocene to early Eocene age. They were collected from the Wadi Tarfa section in the North Eastern Desert. The stratigraphic boundaries of the studied section have been determined using marker planktonic foraminifera. The studied section yielded well-diversified ostracod faunas that appear on the distribution chart (Fig. 2). The recorded species within the studied section exhibit varying stratigraphic ranges, clearly indicating temporal differences. Based on this stratigraphic variation, three local zones can be identified as follows, from older to younger:

*Doricythereis jordanica jordanica* Zone: The base and top of this zone are defined depending on the first occurrence (FO) of the nominate taxon and the FO of *Cytheropteron toshkaensis*, respectively. The defining datum for the base of this zone, the first occurrence of *Doricythereis jordanica jordanica*, is known to occur in the Danian^9,45^.This zone covers about 5 m within the lower portion of the Dakhla Formation in the examined section (Fig. 2). Among the most frequently encountered species in this zone are *Ordoniya bulaqensis*, *Cytherella piacabucuensis farafraensis*, *Xestoleberis kenawyi*, *Bairdia ilaroensis*, *Parakrithe crolifa*, *Krithe echolsae*, *Acanthocythereis denticulata*, *Cytherelloidea attiyaensis* and *Ordoniya ordoniya*. This zone correlates with the *Trachyleberis teiskotensis* Zone of Shahin and El-Nady^46^ from northeastern Sinai, the H1a Subzone of Sarr^24^ from Western Senegal, the lowermost part of the *Paracosta parakefensis*-*Mauritsina coronata* Zone of Ismail and Ied^14^ from Safaga area and the lower part of the *Mauritsina teiskotensis*–*Ordoniya ordoniya* Zone of Shahin^18^ and Shahin and El Baz^13^ from Sinai, Egypt (Table 1). It covers the early Paleocene (Danian) planktonic foraminiferal zones P1c and P2 (Fig. 2).

**Table 1:**
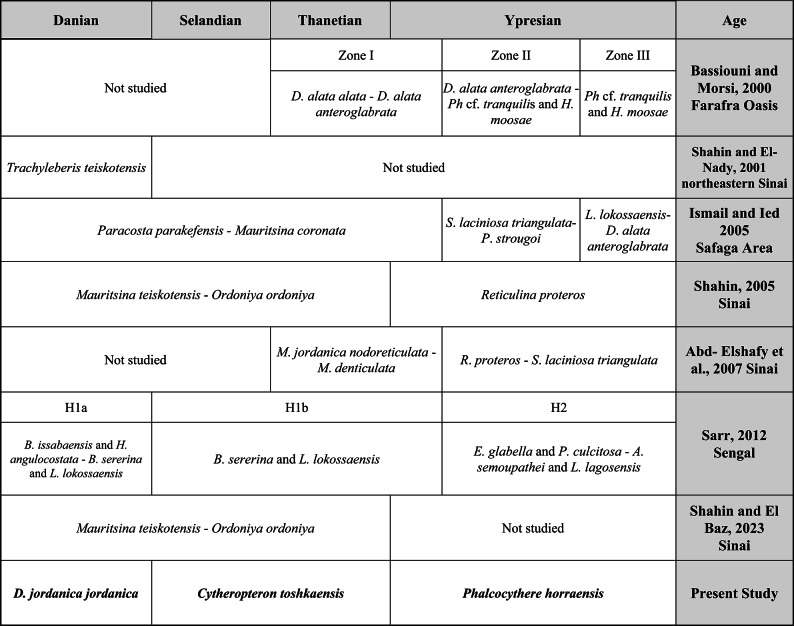
Comparison of the proposed Paleocene-early Eocene ostracod zonal scheme with those from other relevant studies.

*Cytheropteron toshkaensis* Zone: This zone is identified by the total range of the nominal species. It attains about 22 m in thickness, including the middle and top portions of the Dakhla Formation, Tarawan Formation, and the lower portion of the Esna Formation (Fig. 2). Common taxa reported in this zone include: *Cytherella piacabucuensis farafraensis*, *Bairdia ilaroensis*, *Doricythereis jordanica jordanica*, *Xestoleberis tunisiensis* and *Protobuntonia nakkadii*. This zone is partially equivalent to the middle and upper portions of the *Paracosta parakefensis*-*Mauritsina coronata* Zone of Ismail and Ied^14^ from Safaga area, the upper part of the *Mauritsina teiskotensis*-*Ordoniya ordoniya* Zone of Shahin^18^ and Shahin and El Baz^13^ from Sinai and the lower and middle parts of the H1b Subzone of Sarr^24^ from Western Senegal. The upper part of the current zone is partially equivalent to the lower interval of Zone I of Bassiouni and Morsi^11^ from Farafra Oasis and the lower part of the *Mauritsina jordanica nodoreticulata*-*Megommatocythere denticulata* Zone of Abd-Elshafy et al.^47^ from Sinai (Table 1). It represents the late Paleocene (Selandian-Thanetian) of planktonic foraminiferal zones P4 and P5. (Fig. 2).

*Phalcocythere horraensis* Zone: This zone is defined from the last occurrence (LO) of *Cytheropteron toshkaensis* at 27 m to the top of the investigated section (Fig. 2). The present zone is designated as the *Phalcocythere horraensis* Zone due to the exclusive presence of this species only in this interval. Among the most frequently encountered species within this zone are *Parakrithe crolifa*, *Doricythereis jordanica jordanica*, *Acanthocythereis denticulata*, *Ordoniya maanensis*, *Phalcocythere horraensis* and *Cytherella dorsodepressa*. The present zone correlates with the *Reticulina proteros* Zone of Shahin^18^ from Sinai, the upper part of Zone I with Zone II and Zone III of Bassiouni and Morsi^11^ from Farafra Oasis, the uppermost part of the *Paracosta parakefensis*-*Mauritsina coronata* Zone with the *Soudanella laciniosa triangulata*-*Protobuntonia strougoi* Zone and the *Leguminocythereis lokossaensis-Dahomeya alata anteroglabrata* Zone of Ismail and Ied^14^ from Safaga area, the upper part of the *Mauritsina jordanica nodoreticulata*-*Megommatocythere denticulata* Zone with the *Reticulina proteros-Soudanella laciniosa triangulata* Zone of Abd-Elshafy et al.^47^ from Sinai and the upper part of the H1b Subzone with the H2 Zone of Sarr^24^ from Western Senegal (Table 1). It represents the early Eocene (Ypresian) of planktonic foraminiferal zones E1, E2, E3, and E5. (Fig. 2).

### Paleobathymetry

#### Ostracod diversity

Ostracods inhabit various marine settings, spanning from inner neritic to abyssal zone. The diversity of ostracods in neritic environments is significantly higher compared to deeper marine settings^9,10,13,48,49^. This disparity can be attributed to the varying nutrients availability, which are more frequent on the neritic and gradually decrease towards the bathyal and abyssal zones^50,51^. However, it is important to note that lower ostracod diversity is also recognized in lagoonal and inner bay environments, where ecological conditions may limit ostracod species abundance and diversity compared to more open neritic settings^52^. In the studied section, the diversity of ostracods follows a clear stratigraphic pattern. It starts with relatively higher diversity scores, with 6–10 species per sample (sp./sa.), during the early Paleocene (Danian) interval, which decreases significantly (1–6 sp./sa.) during the late Paleocene (Selandian-Thanetian). The early Eocene exhibits a moderately diversified ostracod assemblage (1–9 sp./sa.) (Fig. 2).

The low recovery of ostracods in many samples (e.g., 1–6 specimens/sample in the Tarawan Formation) introduces uncertainty into species richness estimates and biofacies interpretations. Low abundances increase the likelihood of undersampling, meaning some taxa present in the environment may not be represented in the fossil record (Fig. 7).

#### Ostracod R-mode clusters

**Fig. 5:**
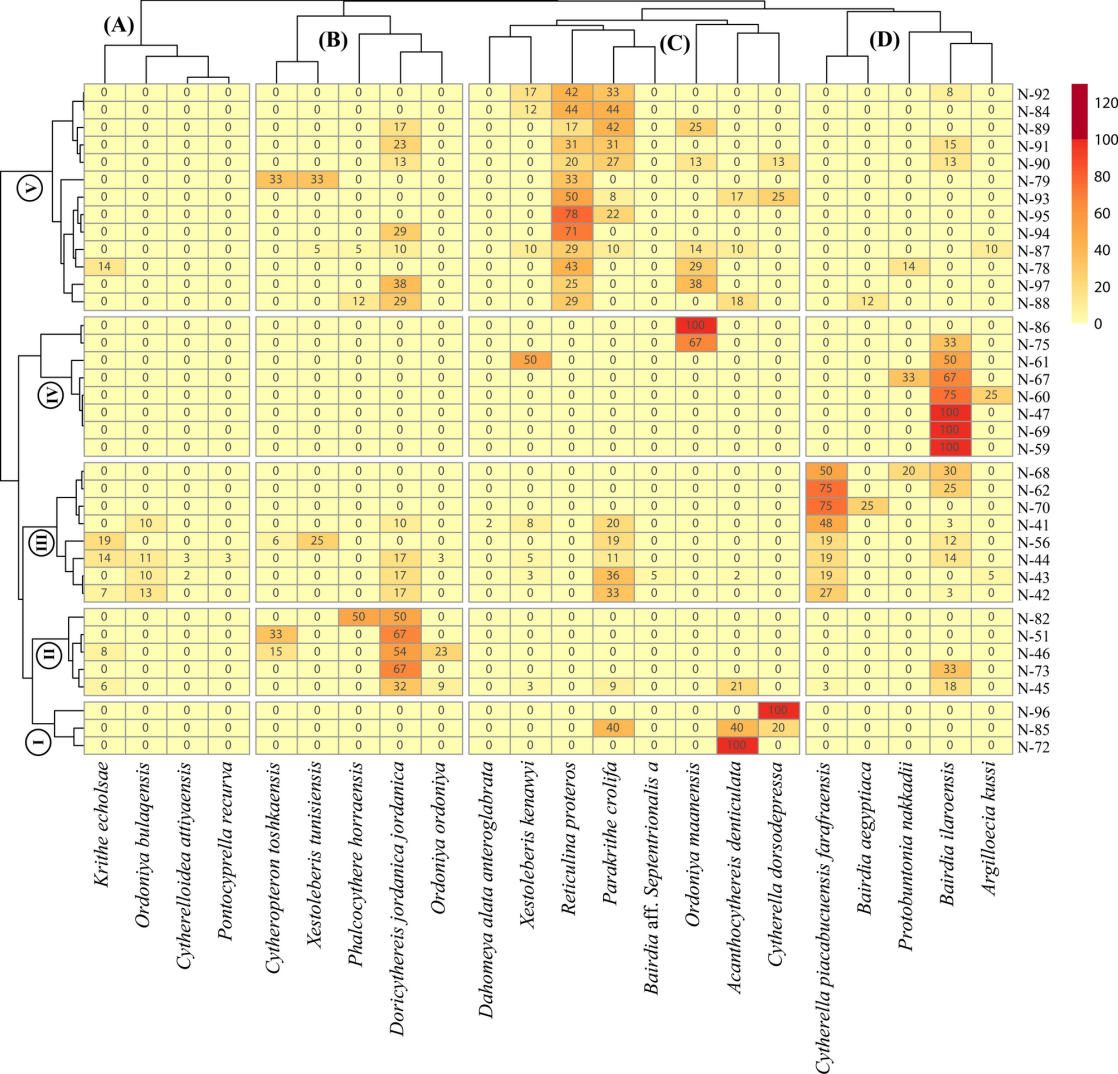
Hierarchical two-way (R-mode and Q-mode) clustering heatmap demonstrating four discrete ostracod clusters and five biofacies across the studied Paleocene-lower Eocene succession.

**Fig. 6:**
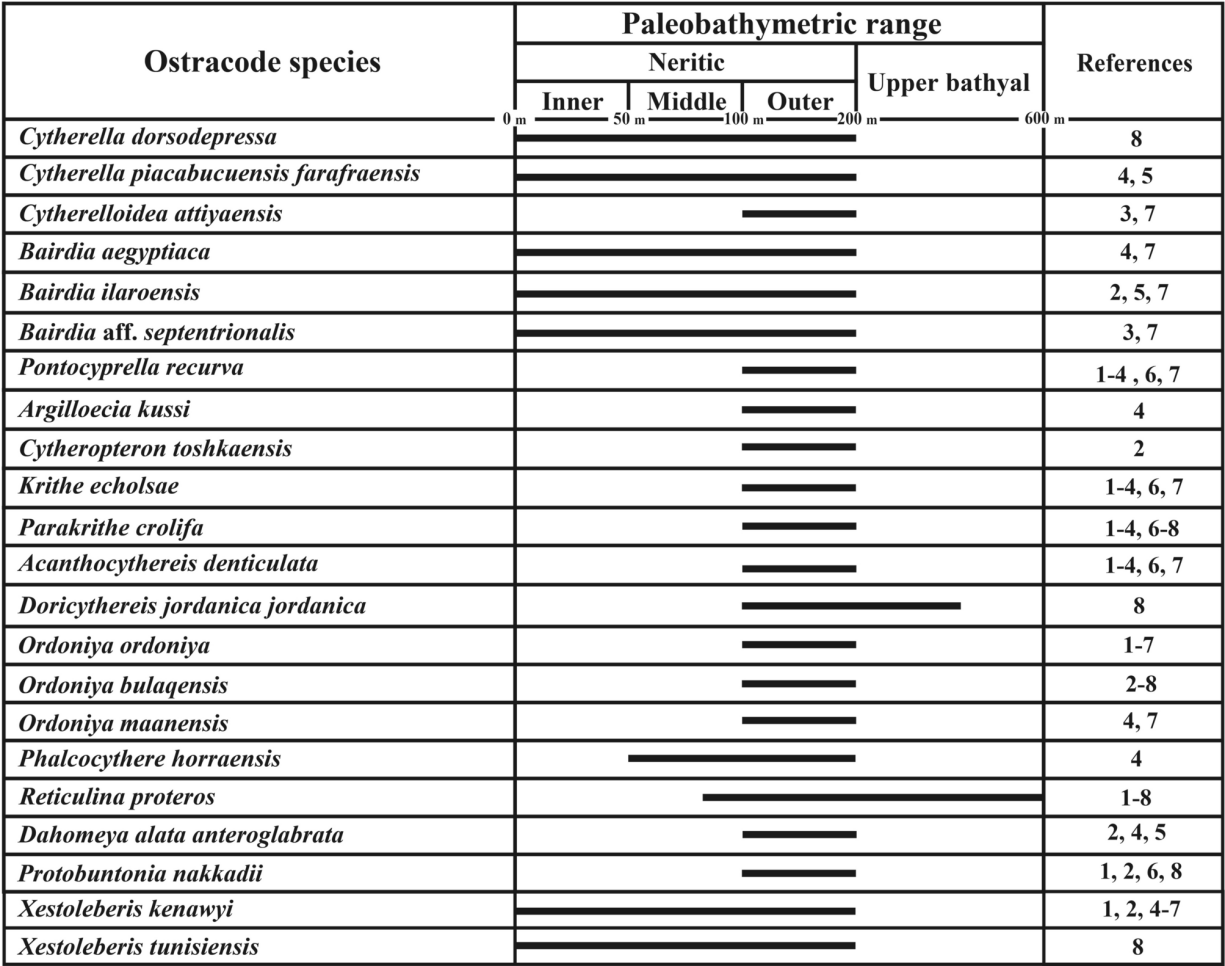
Paleobathymetric range of the recorded ostracod species. Bathymetric assignments are collected from: 1-Donze et al.^16^; 2-Bassiouni and Luger^10^; 3-Morsi^7^; 4-Bassiouni and Morsi^11^; 5-morsi and Speijer^17^; 6- Van Itterbeeck et al.^19^; 7-Morsi and Scheibner^27^; 8-Hewaidy et al.^9^.

The ostracod taxa were categorized into four distinct clusters (A to D) using R-mode cluster analysis (Fig. 5). Each cluster exhibited a unique faunal composition that often reflected different perspectives on the paleoenvironment. Numerous authors have established correlations between ostracod assemblages and depositional depths^7,8,10,11,14,16,17,19,27,53,54^ (Fig. 6). The following four clusters are illustrated:

Cluster A: This cluster combines the faunal content of *Krithe echolsae*, *Ordoniya bulaqensis*, *Cytherelloidea attiyaensis,* and *Pontocyprella recurva* (Fig. 5). This assemblage dominates in the basal part of the studied section and constitutes about 9% of the total ostracod content. The assemblages of this cluster indicate an outer neritic setting (Fig. 6).

Cluster B: It contains several taxa such as *Cytheropteron toshkaensis*, *Xestoleberis tunisiensis*, *Phalcocythere horraensis*, *Doricythereis jordanica jordanica* and *Ordoniya ordoniya* (Fig. 5)*.* These taxa are dispersed through the studied section, particularly in the lower and upper parts, and account for approximately 20% of the total ostracod assemblage. Depending on its faunal content, this cluster denotes an outer neritic-upper bathyal setting (Fig. 6).

Cluster C: It is characterized by an abundant and well-diversified assemblage (Fig. 2). Dominant taxa in this cluster include *Dahomeya alata anteroglabrata*, *Xestoleberis kenawyi*, *Reticulina proteros*, *Parakrithe crolifa*, *Bairdia* aff. *Septentrionalis*, *Ordoniya maanensis*, *Acanthocythereis denticulate* and *Cytherella dorsodepressa* (Fig. 5) This assemblage is represented all over the studys section with increased relative abundance (44%) in the lower and upper parts. This assemblage likely specifies an outer neritic setting (Fig. 6).

Cluster D: The ostracod fauna of this cluster comprises *Cytherella piacabucuensis farafraensis*, *Bairdia aegyptiaca*, *Protobuntonia nakkadii*, *Bairdia ilaroensis* and *Argilloecia kussi* (Fig. 5). The assemblage of this cluster is dispersed primarily in the lower and middle portions of the study section, representing about 27% of the total ostracod fauna. Most of these fauna belong to *Cytherella* and *Bairdia* genera, which are known to inhabit an extensive depth range of marine environments from inner neritic to bathyal settings (Fig. 6).

#### Ostracod biofacies

The Q-mode cluster analysis grouped the samples of the studied section into five biofacies (I–V) according to the composition and structure of ostracod assemblage content (Fig. 5). Based on the depth range of ostracod species, each of these biofacies demonstrates a distinct bathymetric zone.

**Fig. 7:**
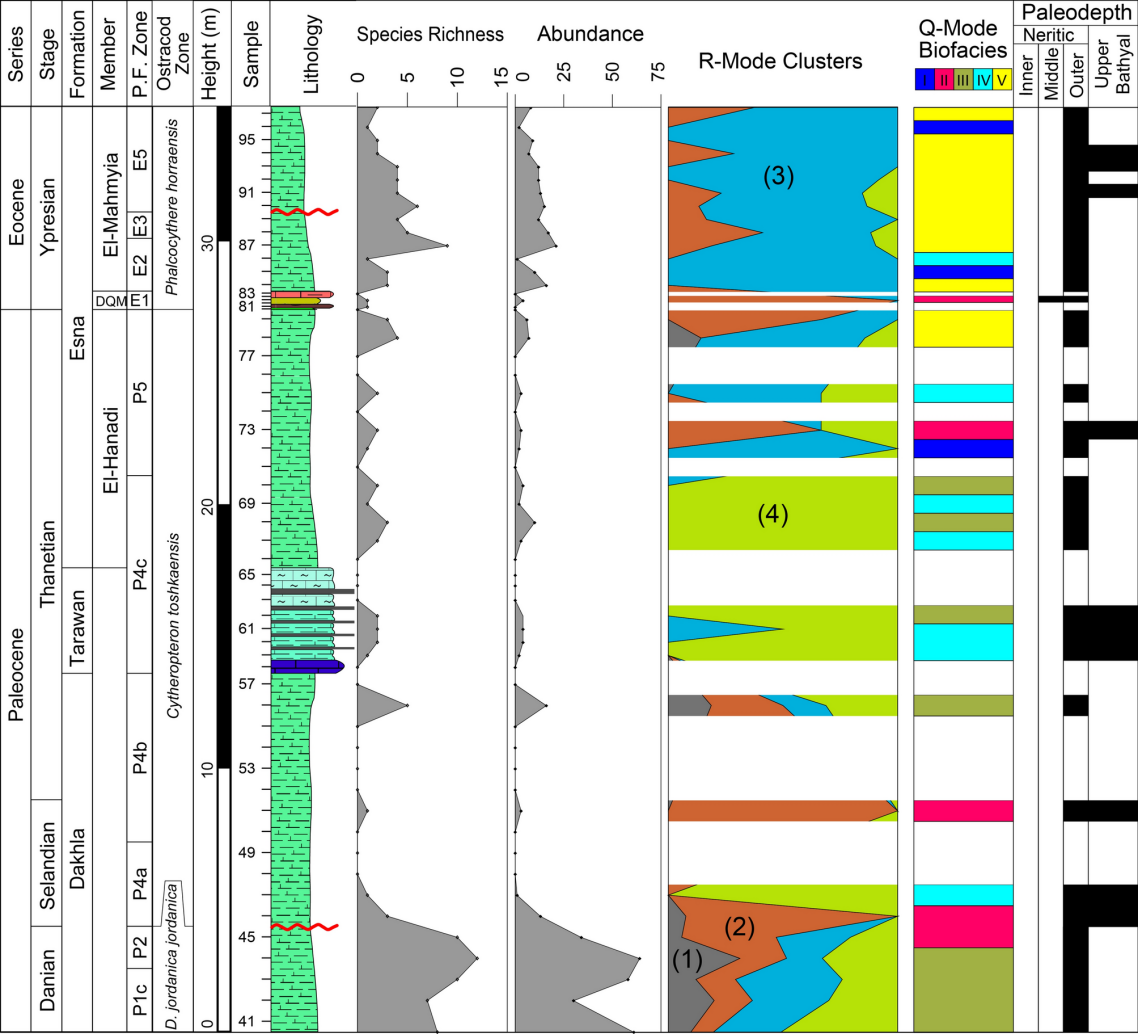
Ostracod-based multivariate and paleoecologic parameters against the Paleocene-early Eocene succession with the paleodepth inferrences.

Biofacies (I): This biofacies comprises three samples (72, 85, and 96) (Fig. 5) within the basal and topmost portions of the Esna Formation. It is characterized by the dominance of faunal cluster C (100%) with a low diversity of only 1–3 species. This biofacies implies deposition in a somewhat outer neritic setting (Fig. 7).

Biofacies (II): This biofacies includes five samples (45, 46, 51, 73, and 82) (Fig. 5) within the Dakhla (middle part) and Esna formations. The ostracod fauna of biofacies II is a mixture of clusters, with approximately 62% of cluster B, 19% of cluster C, 14% of cluster D and 5% of cluster A. The diversity of this biofacies is differentiated from 1 to 8 species. Therefore, it suggests deposition in a deeper outer neritic setting (Fig. 7).

Biofacies (III): It occurs in two intervals within the lower and middle portions of the section studied; the lower part encompasses five samples (41–44 and 56) (Fig. 5) representing the lowest and upper portions of the Dakhla Formation, while the middle part comprises three samples (62, 68 and 70) (Fig. 5) representing the middle portion of the Tarawan Formation and the lowermost Esna Formation. The ostracod diversity in this biofacies varies from 2–3 species in the Tarawan and Esna formations to 6–10 species in the Dakhla Formation (Fig. 2). In this biofacies, the ostracod fauna comprises mixed clusters, with approximately 40% of cluster D, 27% of cluster C, 16% of cluster B, and 5% of cluster A. These characteristics reflect an outer neritic setting in the lower and middle portions of the studied section (Fig. 7).

Biofacies (IV): This biofacies is represented by eight samples (47, 59–61, 67, 69, 78 and 86) (Fig. 5) located in the middle portion of the Dakhla Formation, the lower and middle portions of the Tarawan and Esna formations. It features the high dominance of faunal cluster D (about 75%) and about 25% of faunal cluster C. The diversity of this biofacies is very low 1–2 species only. These features indicate deposition under an outer neritic setting (Fig. 7).

Biofacies (V): It is represented by 13 samples (78, 79, 84, 87–95, and 97) (Fig. 5) within the middle and upper parts of the Esna Formation. It exhibits uneven diversity of 2–9 species in this interval. The ostracod fauna in this biofacies comprises mixed clusters with higher dominance of cluster C (76%) and cluster B (17%). These features refer to an outer setting for this biofacies (Fig. 7).

### Paleobiogeography

**Fig. 8:**
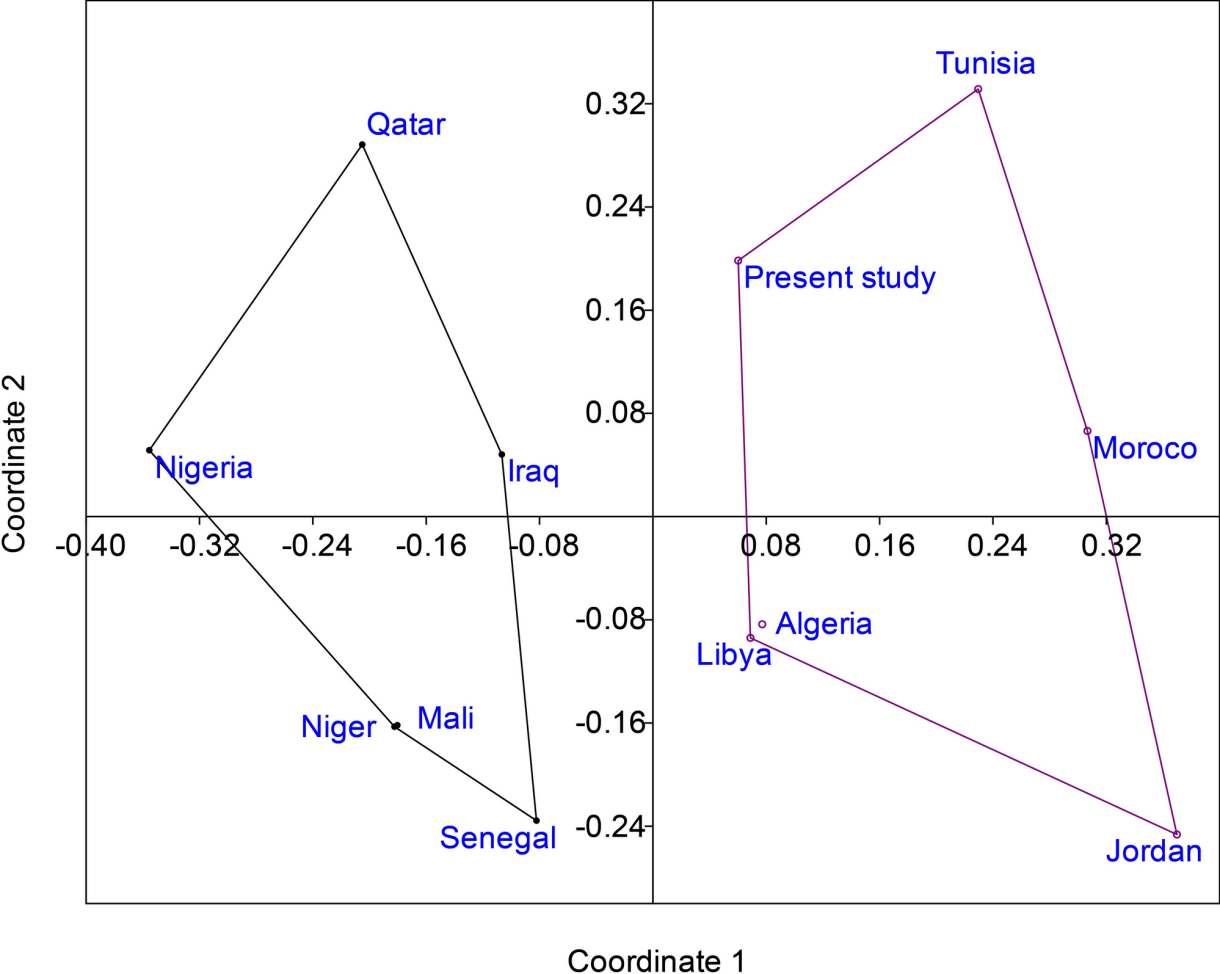
NMDS plot showing the paleobiogeographic relationships of ostracod assemblages from the present study (Wadi Tarfa, Egypt) compared with other regions, including North Africa, West Africa, and the Middle East. The ordination exhibits a stress value of 0.2256, with the first and second axes explaining 36.7% (R^2^ = 0.3667) and 27.8% (R^2^ = 0.2784) of the variance, respectively.

The NMDS analysis (Correlation similarity measure) offered a visual representation of the paleobiogeographic relationships comparing the present study with other localities. The NMDS plot revealed distinct clustering patterns offering significant insights into the distribution and connectivity of ostracod faunas during the Paleocene-early Eocene time. Despite a stress value of 0.2256, which indicates limitations in fully resolving the faunal associations, the ordination highlights strong similarities between southern Tethyan margin localities (Egypt, Tunisia, Morocco, Libya, Algeria, and Jordan). West African and Middle Eastern regions (Qatar, Nigeria, Iraq, Mali, and Senegal) show greater faunal differentiation. The first and second NMDS axes explain 36.7% (R^2^ = 0.3667) and 27.8% (R^2^ = 0.2784) of the variance, respectively, underscoring that while key biogeographic trends are discernible, the ordination failed to fully account for the data complexity. The NMDS plot also reveals potential gradients in ostracod distribution (Fig. 8).

The right side of the generated plot includes the present study, Tunisia, Morocco, Libya, Algeria, and Jordan, suggesting strong faunal similarities among these southern Tethys margin localities. In contrast, the left side of the NMDS plot comprises Qatar, Nigeria, Iraq, Mali, and Senegal (Fig. 8). Greater faunal differences among these regions and from the southern Tethys group, suggesting potential barriers or environmental differences separating the West African and some Middle Eastern faunas from the southern Tethys assemblages. Notably, while geographically representing a part of the Middle East, Jordan clusters with the North African sites, implying a stronger faunal affinity with the southern Tethys margin and suggesting a biogeographic connection between the Levant and North Africa.

Among the ostracod species identified in this study, some exhibit a high degree of endemism, while others demonstrate wider dispersal patterns, connecting them to other locations, including the Middle East and North and West Africa. Out of the 22 species retrieved from the present section, 7 species, *Cytherella dorsodepressa*, *Bairdia* aff. *septentrionalis*, *Cytherelloidea attiyaensis*, *Argilloecia kussi*, *Cytheropteron toshkaensis*, *Phalcocythere horraensis*, *Xestoleberis kenawyi*, are endemic to Egypt^7,8,10–13,17,18,27,55–59^. The remaining 15 were previously documented from other countries of the North and West Africa, as well as the Middle East (Table 2).

**Table 2:** Paleobiogragraphical distribution of the documented ostracod taxa in and outside Egypt.

Species	Mali	Niger	Senegal	Nigeria	Algeria	Tunisia	Moroco	Libya	Jordan	Iraq	Qatar	Present study
*Cytherella dorsodepressa*												*
*Cytherella piacabucuensis farafraensis*						*						*
*Cytherelloidea attiyaensis*												*
*Bairdia aegyptiaca*						*						*
*Bairdia ilaroensis*	*	*	*	*	*			*		*		*
*Bairdia* aff. *septentrionalis*												*
*Pontocyprella recurva*					*	*	*	*		*		*
*Argilloecia kussi*												*
*Cytheropteron toshkaensis*												*
*Krithe echolsae*					*	*				*		*
*Parakrithe crolifa*				*		*				*	*	*
*Acanthocythereis denticulata*					*	*						*
*Doricythereis jordanica jordanica*									*			*
*Ordoniya ordoniya*						*	*		*			*
*Ordoniya bulaqensis*				*		*						*
*Ordoniya maanensis*									*			*
*Phalcocythere horraensis*												*
*Reticulina proteros*			*		*	*			*			*
*Dahomeya alata anteroglabrata*				*								*
*Protobuntonia nakkadii*					*	*		*	*			*
*Xestoleberis kenawyi*												*
*Xestoleberis tunisiensis*				*	*	*						*

Fourteen of the current ostracod species and subspecies were previously documented in various parts of North Africa: 3 species were recorded from Libya, *Bairdia ilaroensis*, *Pontocyprella recurva* and *Protobuntonia nakkadii*^60,61^, 11 species and subspecies were reported from Tunisia, *Cytherella piacabucuensis farafraensis*, *Bairdia aegyptiaca, Krithe echolsae*, *Pontocyprella recurva*, *Parakrithe crolifa*, *Ordoniya ordoniya*, *Ordoniya bulaqensis*, *Reticulina proteros*, *Acanthocythereis denticulata*, *Protobuntonia nakkadii* and *Xestoleberis tunisiensis*^16,19,62–65^, 7 species were documented from Algeria, *Bairdia ilaroensis*, *Krithe echolsae*, *Pontocyprella recurva*, *Reticulina proteros*, *Acanthocythereis denticulata*, *Protobuntonia nakkadii* and *Xestoleberis tunisiensis*^21,66,67^ and 2 species have been recorded from Morocco, *Pontocyprella recurva* and *Ordoniya ordoniya*^20^. Nine ostracod species and subspecies recognized in the current section are also known from other Middle Eastern locales, 5 species and subspecies have been recorded in Jordan, *Doricythereis jordanica jordanica*, *Ordoniya maanensis*, *Ordoniya ordoniya*, *Reticulina proteros* and *Protobuntonia nakkadii*^45,68^, 4 species were found in Iraq, *Bairdia ilaroensis*, *Pontocyprella recurva*, *Krithe echolsae* and *Parakrithe crolifa*^69,70^, one species were recorded from Qatar *Parakrithe crolifa*^71^ (Table 2)*.*

Six ostracod species and subspecies recorded in the present section and known from West African localities, 5 species and subspecies were found in Nigeria, *Bairdia ilaroensis*, *Parakrithe crolifa*, *Ordoniya bulaqensis*, *Dahomeya alata anteroglabrata* and *Xestoleberis tunisiensis*^26,72–76^, 2 species were reported from Senegal, *Bairdia ilaroensis* and *Reticulina proteros*^22,23,77^ and one species *Bairdia ilaroensis* that found in Niger and Mali^25,78^ (Table 2).

## Discussion

### Biostratigraphic implication

A total of 57 samples, representing 22 distinct ostracod species and subspecies, have been obtained from the studied Lower Paleogene rocks in the Wadi Tarfa section in the North Eastern Desert of Egypt. Among these, 5 taxa (22.8%) are exclusively found in the Danian, one taxon occurs solely (4.5%) in the Danian-Selandian, 2 taxa (9.1%) are present in the Danian-Thanetian, one taxon (4.5%) is restricted to the Selandian-Thanetian, one taxon also (4.5%) occurs in the Thanetian, 6 taxa (27.3%) span the Danian-Ypresian, 4 taxa (18.2%) occur in the Thanetian-Ypresian and two taxa (9.1) are exclusively observed in the Ypresian. Notably, no taxa are restricted solely in the Selandian (Fig. 2). There are differences in the recorded ostracod zones during the Paleocene and early Eocene between authors within Egypt and those from outside. For instance, the base and top of the proposed *Doricythereis jordanica jordanica* Zone are identified by the FO of *D. jordanica jordanica* and the FO of *C. toshkaensis*, respectively, whereas the equivalent zone *Trachyleberis teiskotensis* Zone of Shahin and El-Nady^46^ is defined by LO of *Cristaeleberis fornicata* with *Rushdisaidina supracretacea* and the FO of *Trachyleberis teiskotensis.* Furthermore, *Phalcocythere horraensis* Zone is characterized by the LO of *Cytheropteron toshkaensis* to the top of the investigated section, whereas the corresponding *Reticulina proteros* Zone, as described by Shahin^18^ is delineated by FO of *Reticulina proteros* and the FO of the *Martinicythereis samalutensis samalutensis* Zone*.* Therefore, establishing standard ostracod zones for the Paleocene-early Eocene is challenging because most of the ostracod taxa recognized in this interval initially appear in the Paleocene and extend throughout the entire Paleocene^9^, with many of them also extending up into the lower Eocene (Table 1).

### Paleobathymetric interpretation

The paleobathymetric interpretation of the aforementioned ostracod assemblages, in conjunction with the associated lithological characters, were utilized to deduce the paleobathymetry of the ostracod-yielding rock units in the investigated sections.

Dakhla Formation: It consists of gray calcareous shale and marl. The Danian interval of the Dakhla Formation features biofacies II and III, with high diversity and high abundance of ostracod taxa dominantly belonging to clusters C and D. These features refer to an outer neritic setting for this part. The Selandian interval of the Dakhla Formation reveals biofacies II and IV. The diversity is low in this part of the section with a high percent of the ostracod taxa (cluster B) that indicates an outer neritic setting. Only one sample yielded ostracod in the Thanetian part of Dakhla Formation, this sample is represented by biofacies III, with medium ostracod diversity and high abundance of the ostracod taxa belonging to clusters B and D. These characteristics indicate an outer neritic setting (Fig. 7).

Tarawan Formation: It consists of white chalk with cherts bands. This formation is represented by biofacies III and IV, low ostracod diversity and scarce ostracod carapaces of species belonging to cluster D. According to these features, this formation was probably deposited under a deep-water setting (Fig. 7). In fact, the ostracod taxa reported from the Tarawan Formation exhibit wide bathymetric range of inner neritic to upper bathyal settings. Therefore, assigning precise paleodepth assignment in the light of our ostracod data is challenging. Alternatively, other studies focusing on the lithofacies and foraminiferal assemblages have consistently indicated that the Tarawan Formation was deposited in an outer neritic to upper bathyal setting in the North Eastern Desert^79,80^. Furthermore, the scarcity of ostracod fauna within the Tarawan Formation may also be linked to its predominantly pelagic nature. In pelagic environments, benthic habitats are limited.

Esna Formation: The Thanetian part of the Esna Formation is represented by the El-Hanadi Member, which is made up of pale green, slightly compact calcareous shale and marl. All biofacies (I–V) represented in this member with low ostracod diversity. The lowermost part of this member features high proportions of the ostracod taxa of cluster D, whereas the middle and upper parts are branded by high proportion of clusters B and C. These features refer to deposition under an outer setting for this member. The Ypresian portion of the Esna Formation is represented by the Dababyia Quarry Member (DQM) and El-Mahmiya Member. Only one sample yielded ostracod in the DQM, this sample represented by biofacies II, with low ostracod diversity and high abundance of cluster B taxa, indicating an outer neritic setting. The El-Mahmiya Member is composed of grey, fissile, calcareous shales. All samples of this member yielded moderately-highly diversified ostracod fauna. Except for three samples represented by biofacies I and IV, this member is represented by biofacies V. It revealed the higher dominance of the cluster C taxa, along with low frequency of clusters B and D. These criteria suggest deposition under outer neritic to upper bathyal settings (Fig. 7).

We can conclude that, the present section was deposited under outer neritic and upper bathyal settings, as Ouda^5^ stated when he suggested an outer neritic setting for Paleocene-lower Eocene section of Wadi Tarfa. On the other hands, Morsi and Scheibner^27^ studied the Paleocene-early Eocene ostracod from Wadi Tarfa and he also suggested a middle to an outer neritic settings for early to early late Paleocene and deeper marine environment for late Paleocene-early Eocene interval in this section.

### Paleobiogeographic interpretation

The paleobiogeographic patterns revealed by this study provide valuable insights regarding the distribution and connectivity of ostracod faunas during the Paleocene-early Eocene in the southern Tethys-West African realm. The close similarity between the ostracod taxa reported in the present study and those found in the southern Tethys margin, including Libya, Tunisia, Algeria, Morocco, and Jordan, and the observed tight clustering in the NMDS plot indicate a well-connected marine realm (Fig. 8) as Damotte^81^ stated. This connectivity was likely facilitated by a continuous shallow shelf along the Northern African margin and relatively stable environmental conditions across the region. The clustering of Jordan with North African sites in the NMDS plot indicates that this connectivity extended to the eastern Mediterranean, potentially through an extensive epicontinental sea. The mechanism for this faunal exchange is reinforced by the paleogeographic reconstructions of Guiraud et al.^33^, who proposed an extensive epicontinental sea covering parts of North Africa and the Levant during the early Paleogene.

In contrast, there is a less resemblance between the Paleocene-early Eocene ostracod fauna documented in our study and their equivalents in the Middle East, including Iraq and Qatar, as well as West African regions, including Senegal, Nigeria, Niger, and Mali (Fig. 8). This difference may be attributed to the absence of a direct connection to the Tethys Sea and different environmental factors.

**Fig. 9:**
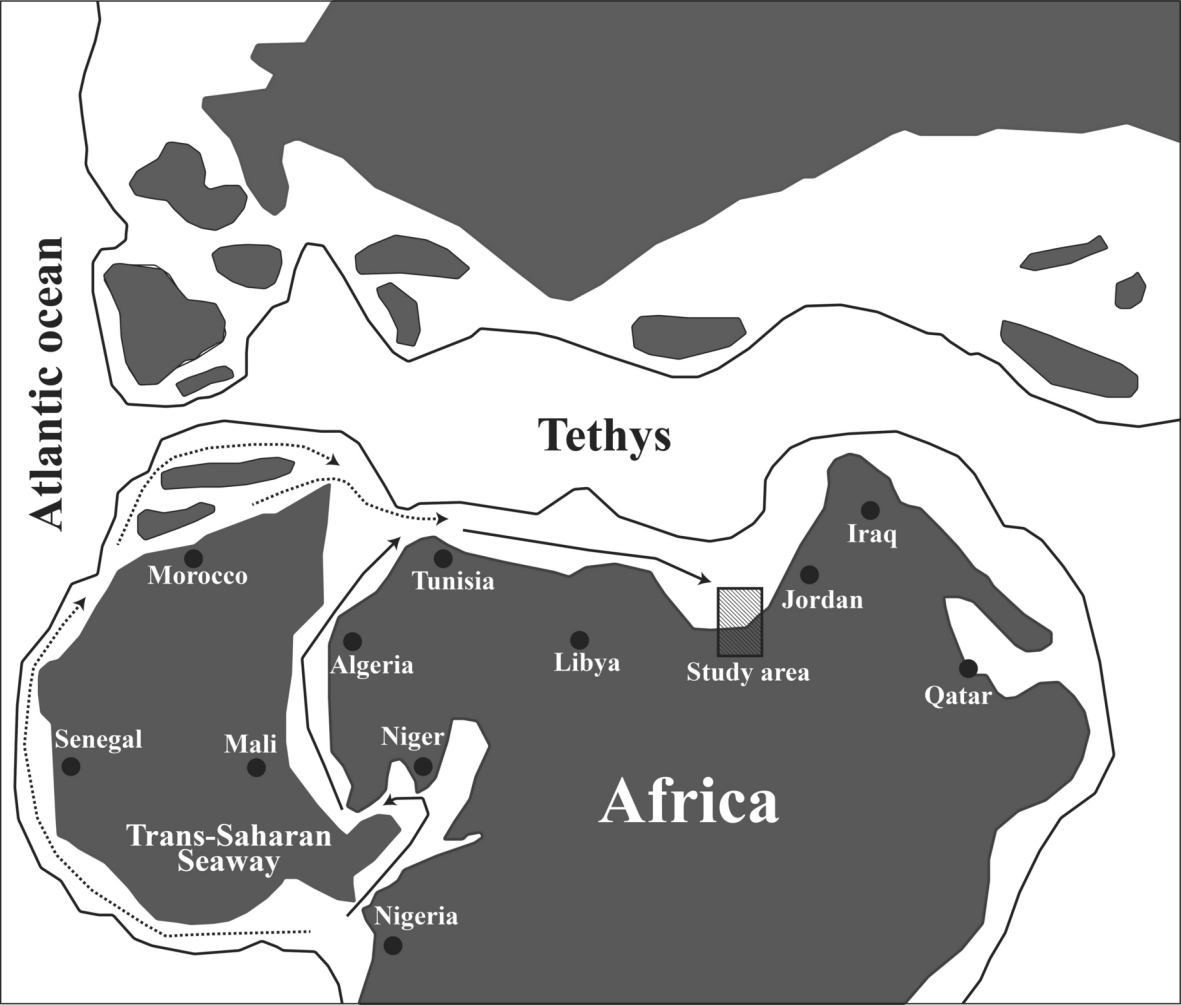
Paleogeographic map demonstrating potential ostracod migration routes from central West African basins to northeast African basins (reprinted after. Morsi et al.^65^ with permission from Elsevier). Dashed arrow denotes West African passage and solid arrow denotes Trans-Saharan passage.

The resemblance between ostracod fauna of the southern Tethys countries and West African basins can be attributed to the migration of ostracods, either via the shallow Trans-Saharan Seaway for epineritic species or through the West African coast for those taxa that dwell in deeper waters^7,9–11,17,61,65,78,82,83^ (Fig. 9). The taxa documented from the West African internal basins (Niger and Mali) most likely migrated through the Trans-Saharan Seaway, while those found in West African coastal basins (Senegal and Nigeria) are assumed to have migrated through the West African coast^9,10,27^ (Fig. 9).

Additionally, the more dispersed clustering of Middle East (except Jordan) and West African sites in the NMDS plot suggests limited but existing marine connections. The unique position of Qatar in the NMDS plot suggests distinct environmental conditions or partial isolation of the Arabian Gulf region during this time (Fig. 8).

However, a limitation in the proposed model arises from the fact that the NMDS ordination exhibits a high stress value (0.2256), with the first and second axes explaining 36.7% (R^2^ = 0.3667) and 27.8% (R^2^ = 0.2784) of the variance, respectively. A significant contributing factor to this limitation is the uneven documentation of ostracod species across regions, with some areas (e.g., West Africa, parts of the Middle East) being poorly studied compared to others (e.g., North Africa, Levant). The lack of comprehensive species records in certain regions may have introduced noise into the analysis, making it difficult to fully resolve the paleobiogeographic relationships. Despite this limitation, the NMDS plot provides valuable insights into the broad patterns of faunal similarity and differentiation across the southern Tethyan margin.

The presence of endemic species (31.8%) in Egypt (Table 2) suggests that despite regional connectivity, some species may be related to local environmental factors, possibly due to unique substrate conditions, local variations in nutrient availability or salinity, and periodic isolation of sub-basins within the broader Tethys realm^33^. This agrees with previous studies from other Egyptian localites, where environmental heterogeneity played a significant role in shaping ostracod distributions^10,11,13,17,55^. The combination of regional connectivity and local environmental factors created conditions conducive to both widespread species distribution and localized endemism.

In conclusion, the ostracod data support a well-connected southern Tethys margin during the Paleocene-early Eocene, with intermittent marine connections between the Tethys and West African basins. Despite the statistical limitations of the NMDS analysis (high stress value), the results suggest potential paleoceanographic barriers or environmental differences separating some Middle Eastern faunas from the southern Tethys assemblages. The high degree of similarity between ostracod faunas along the south Tethyan margin, including the Levant, supports the existence of a continuous shallow marine shelf facilitating faunal exchange. However, the presence of endemic species underscores the importance of local environmental conditions in creating unique ecological niches within this broader connected region.

## Conclusion

The ostracod fauna extracted from the studied Wadi Tarfa section yielded 22 species and subspecies belonging to 16 genera and 8 families. Three zones (*Doricythereis jordanica jordanica* Zone, *Cytheropteron toshkaensis* Zone, and *Phalcocythere horraensis* Zone) were established according to the stratigraphic variation of ostracod fauna across the studied section. However, correlating these local zones with corresponding zones from other regions, both within and outside Egypt, proved difficult because most of the ostracod taxa recorded in this interval have long and paleoenvironmently-controlled stratigraphic ranges. Therefore, the establishment of standardized ostracod zones is problematic. The distribution, abundance, and species richness of the present ostracod assemblages were employed to analyze the paleobathymetry of the studied Paleocene-early Eocene interval. Our findings specify that the studied section was mainly deposited in outer neritic-upper bathyal conditions. Paleobiogeographically, the ostracod fauna reported in the present study show a notable strong resemblance with those found in the southern Tethys margin, including Libya, Tunisia, Algeria, Morocco, and Jordan. In contrast, there is a lesser degree of similarity between the present ostracod fauna and their counterparts in the Middle East, including Iraq and Qatar, as well as West African regions, including Senegal, Nigeria, Niger, and Mali.

## Supplementary Information

Supplementary Information.

## Data Availability

The datasets used and/or analyzed during the current study are available from the corresponding author upon reasonable request.
