# Supplementary material for: Paleoenvironmental and paleobiogeographical significance of Paleocene – early Eocene ostracods in Wadi Tarfa, North Eastern Desert, Egypt
Source: Sci Rep. 2025 Feb 25;15:6828. doi: 10.1038/s41598-025-89560-6 (PMC11862012; doi:10.1038/s41598-025-89560-6)
Supplement: Supplementary file 1 — Supplementary Information. [file 41598_2025_89560_MOESM1_ESM.docx]

**Taxonomic list of the recorded ostracod fauna**

The ostracod fauna examined in this study has been taxonomically categorized into 22 species and subspecies belonged to 16 genera and 8 families. The classification framework employed follows the guidelines set by Horne^41^. Generic assignments primarily adhere to Moore^42^, with later-established genera considered based on the proposals of their respective authors.

Class: **OSTRACODA** Latrielle, 1806

Subclass: **PODOCOPA** Müller, 1894

Order: **PLATYCOPIDA** Sars, 1866

Suborder: **PLATYCOPINA** Sars, 1866

Superfamily: **CYTHERELLOIDEA** Sars, 1866

Family: **CYTHERELLIDAE** Sars, 1866

Genus: ***Cytherella*** Jones, 1849

***Cytherella dorsodepressa*** Morsi, Hewaidy and Samir, 2019

***Cytherella piacabucuensis farafraensis*** Bassiouni and Morsi, 2000

Genus: ***Cytherelloidea*** Alexander, 1929

***Cytherelloidea attiyaensis*** Morsi, 1999

Suborder: **PODOCOPINA** Sars, 1866

Superfamily: **BAIRDIOIDEA** Sars, 1866

Family: **BAIRDIIDAE** Sars, 1866

Genus: ***Bairdia*** Mc’Coy, 1844

***Bairdia aegyptiaca*** Bassiouni and Morsi, 2000

***Bairdia ilaroensis*** Reyment and Reyment, 1959

***Bairdia* aff. *septentrionalis*** Bonnema, 1941

Family: **PONTOCYPRIDIDAE** Müller, 1894

Genus: ***Pontocyprella*** Lyubimova, 1955

***Pontocyprella recurva*** *Esker, 1968*

Genus: ***Argilloecia*** Sars, 1866

***Argilloecia kussi*** Bassiouni and Morsi, 2000

Family: **CYTHERURIDAE** Mueller, 1894

Genus: ***Cytheropteron*** Sars, 1866

***Cytheropteron toshkaensis*** Bassiouni and Luger, 1990

Family: **KRITHIDAE** Mandelstam, 1958

Subfamily: **KRITHINAE** Mandelstam, 1958

Genus: ***Krithe*** Brady, Crosskey and Robertson, 1874

***Krithe echolsae*** Esker, 1968

Genus: ***Parakrithe*** Van Den Bold, 1958

***Parakrithe crolifa*** Bassiouni and Luger, 1990

Family: **TRACHYLEBERIDIDAE** Sylvester-Bradley, 1948

Subfamily: **TRACHYLEBERIDINAE** Sylvester-Bradley, 1948

Genus: ***Acanthocythereis*** Howe, 1963

***Acanthocythereis* *denticulata*** Esker, 1968

Genus: ***Doricythereis*** Gründel, 1976

***Doricythereis jordanica*** (Bassiouni, 1970)

***Doricythereis jordanica jordanica*** (Bassiouni, 1970)

Genus: ***Ordoniya*** Al-Sheikhly, 1985

***Ordoniya ordoniya*** (Bassiouni, 1970)

***Ordoniya bulaqensis*** Bassiouni and Luger, 1990

***Ordoniya maanensis*** (Bassiouni, 1970)

Genus: ***Phalcocythere*** Siddiqui, 1971

***Phalcocythere horraensis*** Bassiouni and Morsi, 2000

Genus: ***Reticulina*** Bassiouni, 1969

***Reticulina proteros*** Bassiouni, 1969

Family: **BRACHYCYTHERIDAE** Puri, 1954

Subfamily: **BRACHYCYTHERINAE** Puri, 1954

Genus: ***Dahomeya*** Apostolescu, 1961

***Dahomeya alata anteroglabrata*** Bassiouni and Luger, 1990

Genus: ***Protobuntonia*** Grékoff, 1954

***Protobuntonia nakkadii*** Bassiouni, 1970

Family: **XESTOLEBERIDIDAE** Sars, 1928

Genus: ***Xestoleberis*** Sars, 1866

***Xestoleberis kenawyi*** Khalifa and Cronin, 1979

***Xestoleberis tunisiensis*** Esker, 1968
